# A novel federated learning framework for medical imaging: Resource‐efficient approach combining PCA with early stopping

**DOI:** 10.1002/mp.18064

**Published:** 2025-09-03

**Authors:** Negin Piran Nanekaran, Eranga Ukwatta

**Affiliations:** ^1^ Department of Engineering University of Guelph Guelph Ontario Canada

**Keywords:** data privacy, data heterogeneity, federated learning, medical image processing, principal component analysis, prostate cancer imaging

## Abstract

**Background:**

Federated learning (FL) facilitates collaborative model training across multiple institutions while preserving privacy by avoiding the sharing of raw data, a critical consideration in medical imaging applications. Despite its potential, FL faces challenges such as high‐dimensional data, heterogeneity among datasets from different centers, and resource constraints, which limit its efficiency and effectiveness in healthcare settings.

**Purpose:**

This study aims to present a novel adaptive FL framework to address the challenges of data heterogeneity and resource constraints in medical imaging. The proposed framework is designed to optimize computational efficiency, enhance training processes, improve model performance, and ensure robustness against non‐independent and identically distributed (non‐IID) data across decentralized data sources.

**Methods:**

The proposed adaptive FL framework addresses the challenges of high‐dimensional data and heterogeneity in nonuniform and decentralized data sources through a key innovation. First, Federated incremental principal component analysis (FIPCA) achieves privacy‐preserving dimensionality reduction by aggregating local scatter matrices and means from participating centers, enabling the computation of a global PCA model. This process ensures data alignment across centers, mitigates heterogeneity, and significantly reduces computational complexity. We evaluated the framework's ability to generalize across institutions in a cross‐site classification task distinguishing clinically significant prostate cancer (csPCa) from non‐csPCa. This assessment used 1500 T2‐weighted (T2W) prostate MRI images from three institutions, where two centers (800 + 350 cases) were used for training and validation, and one center (350 cases) served as an independent test site.

**Results:**

The proposed method significantly reduced the number of global training rounds from 200 to 38, achieving a 98% reduction in energy consumption compared to the standard FedAvg algorithm. The effective use of FIPCA for dimensionality reduction enhanced generalizability, while adaptive early stopping prevented overfitting, leading to an improvement in model performance, with the area under the curve (AUC) on the unseen test center increasing from 0.68 to 0.73 (95 % CI 0.70 – 0.77) on the test center's data. Additionally, the method demonstrated improved sensitivity and specificity, indicating superior classification performance. The integration of FIPCA accelerated convergence by reducing data dimensionality, while the adaptive early‐stopping mechanism further optimized resource utilization and prevented overfitting.

**Conclusions:**

Our adaptive FL approach efficiently handles large, heterogeneous medical imaging data, reducing training time and computational overhead, while improving model accuracy. The substantial reduction in energy consumption and accelerated convergence make it suitable for real‐world healthcare settings.

## 1 INTRODUCTION

AI‐driven models have achieved advances in medical imaging analysis, enhancing patient outcomes, and improving the efficiency of diagnostic processes across various imaging modalities. However, realizing this potential hinges on access to high‐quality, large, and diverse training datasets.^1^ Federated learning (FL) has emerged as a promising solution to this challenge, particularly in the healthcare sector, where data privacy is a critical concern.^2^, ^3^ Traditional machine learning approaches rely on centralized data collection, which poses compliance challenges with data protection regulations such as the General Data Protection Regulation (GDPR) and the Health Insurance Portability and Accountability Act (HIPAA). FL addresses these limitations by enabling decentralized learning across multiple institutions. This approach ensures that sensitive data remains securely localized at each client site while facilitating collaborative model training.^4^

FL introduced by McMahan et al. in 2017,^5^ enables decentralized machine learning by aggregating local model updates through the federated averaging (FedAvg) algorithm, eliminating the need to share raw data. While FedAvg remains a benchmark for performance comparisons,^6^ its effectiveness diminishes in non‐independent and identically distributed (non‐IID) data scenarios, which are prevalent in medical settings. Moreover, it often requires numerous global training rounds to converge, escalating computational costs and increasing the risk of overfitting.^7^ The inherent diversity of medical data—spanning various modalities, dimensions, and feature types—is further compounded by differences in acquisition protocols, equipment, and demographic factors across institutions. This heterogeneity significantly impacts FL model convergence and accuracy, posing a critical challenge in decentralized FL environments.^8^

Techniques like FedMax and Federated disentanglement (FedDis) have been developed to tackle non‐IID data issues. FedMax reduces activation divergence across sources, while FedDis focuses on disentangling anatomical features, leveraging the consistency of anatomical structures across patients to improve anomaly detection and address data heterogeneity.^9^, ^10^, ^11^. Additionally, FedProx^12^ extends FedAvg by introducing a proximal term to reduce model divergence in heterogeneous data, enhancing alignment across local models. Similarly, HarmoFL^13^ addresses data heterogeneity through amplitude normalization, outperforming SOTA methods in tasks such as breast cancer histology, nuclei segmentation, and prostate MRI.

Beyond these, optimization‐based methods such as SCAFFOLD (control variates),^14^ MOON (contrastive learning),^15^ FedDyn (dynamic regularization),^16^ and AFL (agnostic updates)^17^ aim to stabilize client updates and align local objectives with the global goal. Complementing these approaches, Personalization strategies like FedPer,^18^ Per‐FedAvg,^19^ pFedMe,^20^ Ditto,^21^ and FedRep^22^ enable client‐specific adaptations by decoupling shared representation learning from personalized heads. Further, clustering‐based approaches including IFCA^23^ and FedEM^24^ assume latent client groupings or model data as a mixture of experts to better serve diverse distributions.

In parallel, several optimization‐based strategies have been proposed to enhance FL performance under non‐IID conditions. FedAvgM introduces a momentum‐based extension to the standard FedAvg, effectively improving convergence stability in heterogeneous environments.^25^ Similarly, adaptive optimization techniques such as FedAdam, FedAdagrad, and FedYogi adapt popular centralized optimizers to the federated setting, allowing for better handling of client drift and communication efficiency.^26^ These methods dynamically adjust learning rates or gradients across rounds to mitigate the impact of data heterogeneity on global model convergence.

Building on these advances, several studies have applied FL directly to medical imaging problems under real‐world non‐IID constraints. For instance, Kades et al.^27^ integrated FL into the Kaapana platform for prostate MRI segmentation, achieving robust cross‐site performance by training nnU‐Net models across hospitals. Alekseenko et al.^28^ proposed a distance‐aware clustering approach for brain and prostate MRI segmentation, improving personalization and generalization. In CT‐based tasks, Darzi et al.^29^ used Vision Transformers in a federated setting to better capture minority patterns in lung CT, while Yang et al.^30^ introduced semi‐supervised FL to segment COVID lesions from partially labeled CT data. SplitAVG^31^ and FedBN^32^ addressed feature shift and distribution skew in x‐ray and fundus imaging. In histopathology, Adnan et al.^33^ demonstrated that FL with differential privacy preserved accuracy under subtype‐skewed whole slide image (WSI) datasets for lung cancer classification. Multi‐modal FL studies, such as Borazjani et al.,^34^ designed architectures accommodating clients with missing modalities, tackling challenges in cancer staging across heterogeneous data sources. Collectively, these works highlight the adaptability of FL in overcoming practical hurdles in real‐world medical imaging, specially under non‐IID constraints.

While FedAvg remains the standard in FL, other distributed optimization methods capable of handling distributional differences are also under research. Other methods like Federated Dropout,^35^ compression‐based FL techniques such as FedSZ,^36^ and FedGAN (which combines FL with Generative Adversarial Networks) offer solutions for managing imbalanced and non‐IID data, particularly in resource‐limited settings like Internet of Things (IoT) and Internet of Medical Things (IoMT).^37^, ^38^ However, despite these innovations, challenges such as communication overhead, data heterogeneity, and bias in model aggregation remain significant.^39^

Our work advances FL by introducing a novel approach that combines federated incremental PCA (FIPCA) and adaptive early stopping mechanisms to address the challenges of data heterogeneity and computational efficiency in medical imaging. While early stopping based on client validation loss aggregation has been previously explored,^40^, ^41^ our contribution lies in how it is harmonized with privacy‐preserving dimensionality reduction to improve convergence speed, generalizability, and energy efficiency in a realistic healthcare setting. To the best of our knowledge, this is the first study to apply FL for dimensionality reduction using FIPCA, leveraging the aggregation of local means and scatter matrices from individual centers to compute a global variance. This approach enables consistent and privacy‐preserving dimensionality reduction across centers with diverse data distributions, effectively aligning these distributions while retaining essential features. By reducing computational complexity and preserving critical variations, FIPCA not only addresses the challenge of heterogeneity but also enhances model performance, as reflected in improved AUC values. Additionally, we incorporate adaptive early stopping mechanisms at both client and server levels to further optimize resource utilization. Client‐side early stopping halts local training when performance improvements plateau, reducing unnecessary computations and preventing overfitting on local datasets. Meanwhile, server‐side adaptive early stopping monitors aggregated client validation losses to determine when global training should cease, ensuring efficiency and preventing overfitting in the global model. Together, these innovations significantly improve training efficiency and robustness to non‐IID data, addressing key challenges in federated learning for high‐dimensional medical data.

## 2 METHODOLOGY

In this study, we describe a method for prostate cancer classification using T2W MRI images, preserving the full 3D context since lesions frequently span multiple slices. We propose a novel FL algorithm that enhances the standard FedAvg, improving both training efficiency and model performance in FL, particularly for medical imaging applications involving high‐dimensional data. This study used 1500 T2W prostate MRI scans from three medical centers. Radboud University Medical Center (RUMC, 800 cases) and Prostate Cancer Neuroendocrine Network (PCNN, 350 cases) provided data for training and validation, while Ziekenhuisgroep Twente (ZGT, 350 cases) served as an independent test site. These data were used to evaluate the generalizability of the proposed framework in a cross‐institution federated classification setting.

**FIGURE 1:**
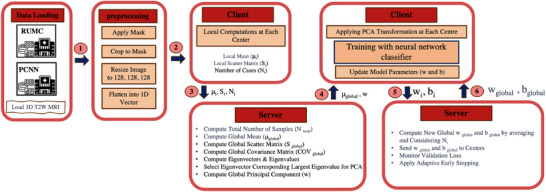
Our proposed adaptive process in a FL setup, illustrating federated data preprocessing, client‐side adaptive training, and server‐side coordination with an early stopping mechanism. Steps 5 and 6 repeat as rounds between clients and the server until the convergence criteria are met. FL, federated learning.

Figure 1 illustrates the complete process. Our adaptive FL pipeline comprises three key components: (i) a novel method based on principal component analysis (PCA) for privacy‐preserving dimensionality reduction and harmonization of inter‐center variation, (ii) client‐side training with a custom loss function that balances penalties for false negatives and false positives while incorporating an AUC‐oriented regularization term, and (iii) an early‐stopping strategy to minimize unnecessary computation and enhances training efficiency. The following sections detail the key algorithms and processes underlying our proposed method.

### 2.1 Preprocessing via FIPCA

FIPCA is designed to harmonize feature distributions across institutions and to project data into a lower‐dimensional space, all while preserving data privacy. In the first step of our preprocessing method, local statistics are computed at each center. Each center calculates its own local mean $\mu_{i}$ and local scatter matrix $S_{i}$ from its data. To compute these statistics, the segmented 3D T2W MRI images ($X_{i}$) are first flattened into voxel‐level vectors. Throughout this paper, the term *feature* refers to this voxel‐intensity vector obtained by flattening the segmented 3D T2W image. This vectorisation transforms each image into a point in a common high‐dimensional space, allowing FIPCA to compute covariance structures across centers. This step is essential, as the scatter matrices used by FIPCA are defined over vectorized feature spaces and must be linearly aggregable. The centers then send these local statistics ($\mu_{i}$, $S_{i}$, and $N_{i}$) to the server for the aggregation, ensuring that no raw data is shared.

In the second step, the global FIPCA is performed at the server. The global covariance matrix is derived by normalizing the aggregated scatter matrix received from all centers. An eigen‐decomposition is then carried out to extract the top k principal components, capturing the most significant directions of variance across the data from all centers. In the third and final step, each center applies the FIPCA model to its local data. By subtracting the global mean and projecting onto the principal components, the data is transformed into a unified, lower‐dimensional space. The transformed datasets are subsequently split into training and testing sets for the federated learning process.

Algorithm 1 outlines the step‐by‐step process of loading, preprocessing, and splitting the data for FL. The number of principal components (k) and the batch size (b) are specified to guide the dimensionality reduction process. The value of (k) can be selected based on desired variance retention or validated empirically. Batching is used solely to reduce memory usage during computation of local scatter matrices; all batches are fully aggregated, and thus batch size does not affect the resulting PCA components or model performance. The output of this algorithm includes the derived FIPCA components (W) and the transformed datasets for each center, which are projected into a lower‐dimensional space for subsequent training.

ALGORITHM 1 **FIPCA for 3D medical images**

**Table:** 

1:	**Input**:
2:	$X_{i}$: Data from center i (in 3D format)
3:	M: Number of centers
4:	$N_{i}$: Number of samples at center i
5:	d: Flattened data dimension ($d={\text{resolution}}^{3}$)
6:	k: Number of principal components
7:	Batch size b
8:	**Output**:
9:	PCA components $W\in R^{d\times k}$
10:	Transformed datasets $X_{train}^{\left(i\right)},X_{test}^{\left(i\right)}$ for each center
11:	**Initialize**:
12:	$S_{global}=0$: Global scatter matrix
13:	$N_{total}=0$: Total number of samples
14:	**Step 1: Local Statistics at Each Center**
15:	**for** each center i=1 to M **do**
16:	Load and flatten $X_{i}$
17:	**for** each batch $B_{i}$ of size b **do**
18:	Compute batch mean: $\mu_{B_{i}}=\frac{1}{b}\sum_{x\in B_{i}}x$
19:	Center data: $X_{B_{i}}^{\prime }=B_{i}-\mu_{B_{i}}$
20:	Update global scatter matrix: $S_{global}+=X_{B_{i}}^{\prime T}X_{B_{i}}^{\prime }$
21:	Update total sample count: $N_{total}+=b$
22:	**end for**
23:	**end for**
24:	**Step 2: Global PCA**
25:	Normalize scatter matrix: $Cov_{global}=\frac{S_{global}}{N_{total}}$
26:	Compute eigenvectors: $W\in R^{d\times k}$ from $Cov_{global}$
27:	**Step 3: Apply PCA to Each Center**
28:	**for** each center i=1 to M **do**
29:	Load $X_{i}$ and subtract global mean: $X_{i}^{\prime }=X_{i}-\mu_{global}$
30:	Apply PCA transformation: $X_{i}^{\prime PCA}=X_{i}^{\prime }W$
31:	Split into training and testing sets and save
32:	**end for**
33:	**End of Algorithm**

This preprocessing method offers several key advantages. First, it maintains privacy by ensuring that no raw data is exchanged between centers. Second, it is efficient, as the FIPCA processes data in memory‐efficient batches. Finally, it ensures consistency by projecting the data from all centers into a unified lower‐dimensional space, mitigating the effects of diverse data distributions across centers.

Given the known heterogeneity in image acquisition across institutions in the PI‐CAI dataset—including differences in MRI vendors, resolution, and csPCa prevalence—site‐specific factors such as scanner type (e.g., Siemens at RUMC vs. Philips at ZGT), resolution settings, and csPCa distribution (29.5% at RUMC, 31.1% at PCNN, and 22.8% at ZGT) introduce statistical non‐IID characteristics. These variations in scanner hardware and image quality impact T2W‐derived features. To mitigate this inter‐site variability, we adopted FIPCA as a central pre‐processing step. This ensured a more aligned and harmonized feature space before training, which was critical for improving model convergence and generalization under federated non‐IID conditions.

### 2.2 Client‐side training

On the client side, a custom loss function is employed to address the challenges posed by class imbalance and to improve classification performance. The loss function includes terms that balance penalties for false negatives and false positives, and incorporates an AUC‐oriented regularization component. This design is particularly important for medical imaging tasks, where both false negatives and false positives can have significant consequences. The loss function is expressed as Equation 1. Where the overall loss function represents the standard cross‐entropy loss, $L_{\text{CE}}$, along with additional terms to account for false negatives, false positives, and AUC‐oriented regularization. The false negative penalty, FN_penalty, is defined as $y_{\text{true}}\cdot \left(1-y_{\text{pred}}\right)$, while the false positive penalty, FP_penalty, is given by $\left(1-y_{\text{true}}\right)\cdot y_{\text{pred}}$. Additionally, the AUC‐oriented regularization term, AUC_reg, is calculated as ${\left(y_{\text{pred}}-y_{\text{true}}\right)}^{2}$. The coefficients $\lambda_{\text{FN}},\lambda_{\text{FP}},\lambda_{\text{AUC}}$ determine the relative weight of each of these components in the final loss function. For the present use case, we set $\lambda_{\text{FN}}=2$, $\lambda_{\text{FP}}=1$, and $\lambda_{\text{AUC}}=0.1$. These values were selected via grid search and reflect the class imbalance (csPCa prevalence ≈28%) by assigning greater penalty to false negatives.

(1) $$\begin{array}{ccc} L_{\text{custom}} & = & L_{\text{CE}}+\lambda_{\text{FN}}\cdot {\text{FN}}_{\text{penalty}}+\lambda_{\text{FP}}\cdot {\text{FP}}_{\text{penalty}}\\ & & +\;\lambda_{\text{AUC}}\cdot {\text{AUC}}_{\text{reg}}\\ & = & L_{\text{CE}}+\lambda_{\text{FN}}\cdot y_{\text{true}}\cdot \left(1-y_{\text{pred}}\right)\\ & & +\;\lambda_{\text{FP}}\cdot \left(1-y_{\text{true}}\right)\cdot y_{\text{pred}}+\lambda_{\text{AUC}}\cdot {\left(y_{\text{pred}}-y_{\text{true}}\right)}^{2} \end{array}$$

The neural network used for client‐side training comprises three fully connected layers. The input layer accepts data with dimensions equal to the number of principal components derived from FIPCA preprocessing. The first dense layer consists of 128 units with ReLU activation, followed by batch normalization. The second dense layer has 64 units, also with ReLU activation and batch normalization. A dropout layer with a rate of 0.5 is applied before the output layer, which contains 2 units with softmax activation for binary classification. The total number of trainable parameters depends on the number of principal components (k) provided by FIPCA. The model is optimized using the Adam optimizer with the dynamically adjusted learning rate based on the server round number t as per Equation 2. Where $lr_{0}$ is the initial learning rate, decay_rate is the decay rate, and decay_steps determines how often the decay is applied (every 25 rounds). Additionally, an early stopping mechanism is implemented to prevent overfitting, which monitors validation loss with a patience of five epochs. Algorithm 2 provides an overview of the client‐side training process.

(2) $$lr=lr_{0}\times {\left(\text{decay}\_\text{rate}\right)}^{\left\lfloor \frac{t}{\text{decay}\_\text{steps}}\right\rfloor }$$

ALGORITHM 2 **Client training with custom loss function and early stopping**

**Table:** 

1:	Initialize seed s←12345
2:	Load $\left(X_{\text{train}},y_{\text{train}}\right),\left(X_{\text{val}},y_{\text{val}}\right)$ from local dataset
3:	Define neural network model Model with specified architecture
4:	Compile Model with optimizer, loss function $L_{\text{CE}}$, and evaluation metrics
5:	Apply class weights to handle class imbalance
6:	Train Model on $X_{\text{train}}$ with early stopping based on validation loss
7:	Save initial training history
8:	**while** training not converged **do**
9:	Receive global weights $w_{g}$ and server round t from the server
10:	Set model weights $w\leftarrow w_{g}$
11:	Compute learning rate lr using Equation 2
12:	Define custom loss function $L_{\text{custom}}$ as in Equation 1
13:	Compile Model with optimizer (learning rate lr), loss function $L_{\text{custom}}$, and metrics
14:	Train Model on $X_{\text{train}}$ with early stopping
15:	Evaluate Model on validation data to obtain $L_{\text{val}}$, $A_{\text{val}}$
16:	Save training history
17:	Send updated weights w, number of samples, and metrics $\left\{L_{\text{val}},A_{\text{val}}\right\}$ to the server
18:	**end while**

### 2.3 Server‐side coordination

The server initializes the global model parameters $w^{\left(0\right)}$ and coordinates the training process over multiple global rounds. At each round t, a subset of clients $S_{t}$ is selected to participate in training. Each client $k\in S_{t}$ trains the global model on its local dataset using the custom loss function and client‐side early stopping, and returns updated local model parameters $w_{k}^{\left(t\right)}$ along with their validation loss $L_{k}^{\left(t\right)}$ and validation accuracy $a_{k}^{\left(t\right)}$.

The server aggregates the local model updates using a weighted average, where the weights are based on both the number of training samples and the clients' validation accuracy, as shown in Equation 3, where $n_{k}=\left|D_{k}\right|$ is the number of training samples on client k, $a_{k}$ is the validation accuracy of client k, and $n=\sum_{k\in S_{t}}n_{k}\cdot a_{k}$ is the total weighted samples across all participating clients. This weighted aggregation gives higher importance to clients with better validation performance, which can improve the overall model convergence.

Then the server computes the aggregated validation loss $L_{t}$ using Equation 4 and applies a practical early stopping mechanism, informed by aggregated client validation losses, to halt training once improvements plateau beyond a defined patience and threshold. While not novel on its own, this mechanism plays a vital role in the overall efficiency of our framework, especially in conjunction with FIPCA. Algorithm 3 outlines the server coordination process.

(3) $$w^{\left(t\right)}=\frac{\sum_{k\in S_{t}}n_{k}\cdot a_{k}\cdot w_{k}^{\left(t\right)}}{\sum_{k\in S_{t}}n_{k}\cdot a_{k}}$$

(4) $$L_{t}=\frac{\sum_{k\in S_{t}}n_{\text{val},k}\cdot L_{k}^{\left(t\right)}}{\sum_{k\in S_{t}}n_{\text{val},k}}$$

ALGORITHM 3 **Federated learning server with adaptive early stopping**

**Table:** 

1:	**Initialize** global weights $w^{\left(0\right)}$, best loss $L_{\text{best}}\leftarrow \infty$, wait counter c←0, patience p, tolerance ε, delta δ, minimum rounds $t_{\min}$
2:	**for** each round t=1 to T **do**
3:	**if** training converged **then**
4:	**Break** (Early stopping)
5:	**end if**
6:	**Select** a subset of clients $S_{t}$
7:	**Broadcast** global weights $w^{\left(t-1\right)}$ and round t to clients in $S_{t}$
8:	**Receive** updated weights $\left\{w_{k}^{\left(t\right)}\right\}$, validation losses $\left\{L_{k}^{\left(t\right)}\right\}$, and validation accuracies $\left\{a_{k}^{\left(t\right)}\right\}$ from clients
9:	**Aggregate** global weights using Equation (6)
10:	**Compute** aggregated validation loss: $L_{t}=\frac{\sum_{k\in S_{t}}n_{\text{val},k}\cdot L_{k}^{\left(t\right)}}{\sum_{k\in S_{t}}n_{\text{val},k}}$
11:	**if** $L_{t}<L_{\text{best}}-\delta$ **then**
12:	$L_{\text{best}}\leftarrow L_{t}$
13:	c←0
14:	**else if** $L_{t}\leq L_{\text{best}}+\varepsilon$ **then**
15:	c←0
16:	**else**
17:	c←c+1
18:	**end if**
19:	**if** $t\geq t_{\min}$ **and** c≥p **then**
20:	**Save** $w^{\left(t\right)}$ as final model
21:	**Break** (Early stopping)
22:	**end if**
23:	**end for**

## 3 RESULTS

We conducted a series of experiments to evaluate the effectiveness of our proposed method. The Prostate Imaging Cancer AI (PI‐CAI) dataset,^42^ consisting of 1500 patient cases, was utilized for this purpose. These cases were sourced from three distinct medical institutions. Data from two centers were used for training and validation, while the third center was exclusively used for testing the model's generalization performance. The distribution of total cases, as well as csPCa cases across the centers, is summarized in Table 1. This setup allowed us to evaluate the method's performance in a realistic federated learning scenario where data is distributed across institutions. To provide further granularity, Table 2 presents a breakdown of each center's data by training, validation, and test set splits, along with corresponding class distributions.

**TABLE 1:** Distribution of total cases and csPCa cases across centers. PCNN and RUMC are used for training and validation, while ZGT is reserved for testing.

Center	Total Cases	csPCa Cases	Purpose
RUMC	800	236	Train/validation
PCNN	350	109	Train/validation
ZGT	350	80	Test
**Total**	**1500**	**425**	

Abbreviations: csPCa, clinically significant prostate cancer; PCNN, Prostate Cancer Neuroendocrine Network; RUMC, Radboud University Medical Center; ZGT, Ziekenhuisgroep Twente.

**TABLE 2:** Distribution of total cases and csPCa cases across centers and dataset splits. Training and validation sets are drawn from RUMC and PCNN. ZGT is reserved for testing.

Center	Set	Total Cases	csPCa Cases	Non‐csPCa Cases
RUMC	Train	640	190	450
	Validation	160	46	114
PCNN	Train	280	87	193
	Validation	70	22	48
ZGT	Test	350	80	270

Abbreviations: csPCa, clinically significant prostate cancer; PCNN, Prostate Cancer Neuroendocrine Network; RUMC, Radboud University Medical Center; ZGT, Ziekenhuisgroep Twente.

**FIGURE 2:**
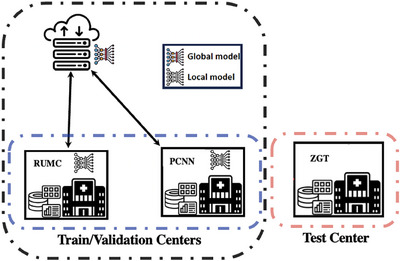
Federated learning topology using the Flower framework with two centers (RUMC and UMCG) for training/validation and one center (ZGT) for testing.

Figure 2 illustrates the FL setup used in this study, with RUMC and PCNN serving as the training and validation centers. The local models are trained separately on these centers and are aggregated on the global server using the Flower framework. The global model is then tested at ZGT to evaluate its generalization performance in an unseen dataset.

The dataset used for the experiments provided 3D prostate segmentation labels for each patient, allowing us to utilize the 3D prostate structure in defining the input space. Feature vectors were obtained by flattening the voxel intensities of the prostate‐segmented 3D T2W images into high‐dimensional vectors, which were then reduced federatively using FIPCA prior to classification. We applied FIPCA to reduce the dimensionality to 10 features, retaining the essential structure of the data while improving computational efficiency and preserving data privacy. Each center computed local scatter matrices and means from its data, and these local statistics were aggregated at the server to derive the global FIPCA model without sharing raw data. The neural network architecture was adjusted to match the input size of the FIPCA‐transformed data, ensuring optimal processing. Multiple clients, each with different local data distributions, were incorporated into the experiment, simulating the heterogeneity commonly found in FL scenarios. To assess the model's convergence and generalization capabilities, validation loss was monitored throughout the global training rounds. But the final evaluation is on test data center which is totally unseen.

**FIGURE 3:**
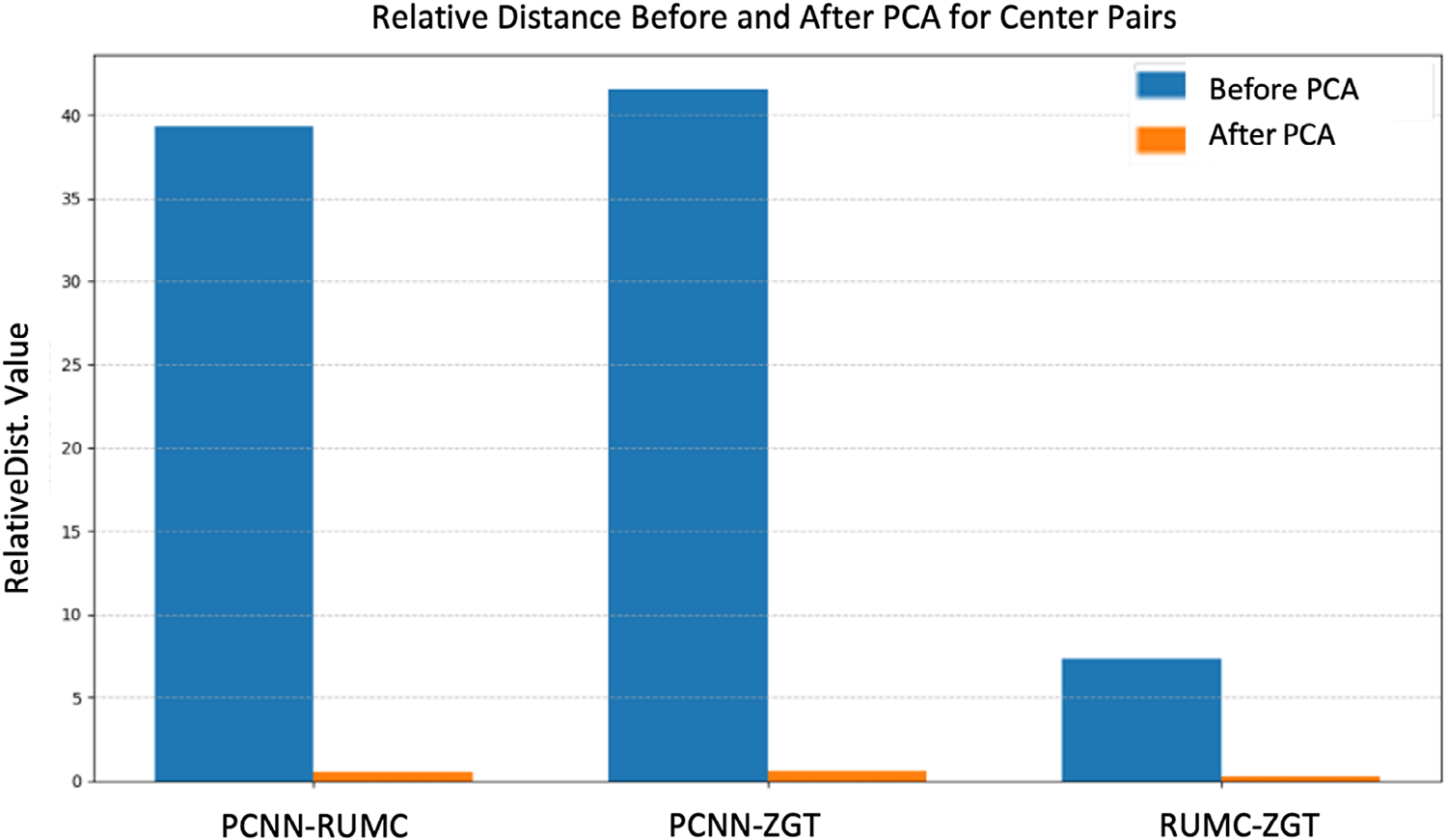
Relative distance between site centroids before and after applying FIPCA. Blue bars represent distances computed on standardized raw features, while orange bars show distances after FIPCA transformation. FIPCA, federated incremental principal component analysis.

To evaluate the impact of FIPCA on cross‐site feature alignment, we computed the relative distance between site centroids before and after dimensionality reduction. Relative distance measures inter‐site separation normalized by each site's internal variability, providing a more interpretable metric of heterogeneity than absolute distances—especially in federated settings. As illustrated in Figure 3, the relative distance decreased dramatically by 96% –99% across all center pairs following the FIPCA transformation. This substantial reduction—observed consistently between PCNN–RUMC, PCNN–ZGT, and RUMC–ZGT—demonstrates effective alignment of site‐level feature distributions. These results confirm that FIPCA not only preserves privacy and reduces dimensionality but also harmonizes feature distributions across institutions, a critical factor for robust federated learning in medical imaging.

**FIGURE 4:**
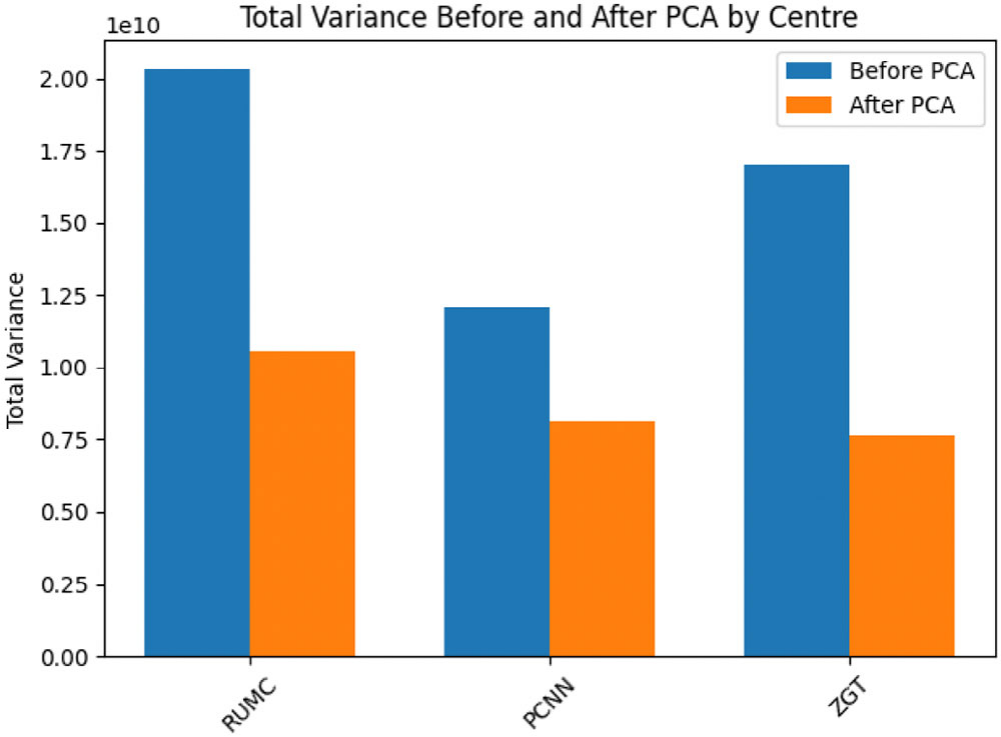
Total variance before and after FIPCA for each center. FIPCA, federated incremental principal component analysis.

The total variance before and after applying FIPCA for each center is also illustrated in Figure 4. The FIPCA approach effectively reduced dimensionality while retaining between 45% and 68% of the total variance across centers. This demonstrates the method's ability to align data distributions across centers, addressing the challenges posed by heterogeneity in FL and enhancing overall model performance by improving consistency. While increasing the number of principal components (PCs) beyond 10 would preserve more variance, it did not yield improvements in the AUC for downstream tasks. Therefore, 10 PCs were selected as an optimal balance between variance retention and computational efficiency.

### 3.1 Efficiency evaluation

Table 3 presents a comparison between the standard FedAvg algorithm and our proposed method. The results indicate that our FL framework substantially reduced the number of global rounds required for convergence while also improving AUC. Specifically, the standard FedAvg algorithm required 200 global rounds to achieve an AUC of 68%. The number of global rounds for FedAvg was set to 200 based on Moradi et al.,^43^ who found this configuration optimal for the PI‐CAI dataset. In contrast, our proposed adaptive approach only required 38 global rounds and achieved a higher AUC of 73%. This demonstrates the effectiveness of the proposed method in enhancing both training speed and model performance.

**TABLE 3:** Performance comparison between standard FedAvg and adaptive early stopping FedAvg.

Method	Global Rounds	AUC on Test center
Standard FedAvg	200	0.68
Our Proposed Method	38	0.73

Abbreviation: FedAvg, federated averaging.

According to Camajori et al.'s findings,^44^ FL models typically range from 30 to 150 MB per learning round. Reducing the rounds from 200 to 38 significantly eases the communication load in large‐scale FL networks. This optimization not only saves computational resources but also prevents overfitting by avoiding unnecessary training rounds.

The value of decentralized learning in FL is further underscored by comparing AUC results from models trained solely on individual centers and evaluated on a different center's test set. For example, a model trained on the PCNN center (one of the training centers) and evaluated on the ZGT test set achieved an AUC of 0.627. Similarly, a model trained on the RUMC center and evaluated on the ZGT test set resulted in a lower AUC of 0.596. These findings highlight the limitations of centralized training on individual centers due to data heterogeneity, where models struggle to generalize effectively across centers.

**FIGURE 5:**
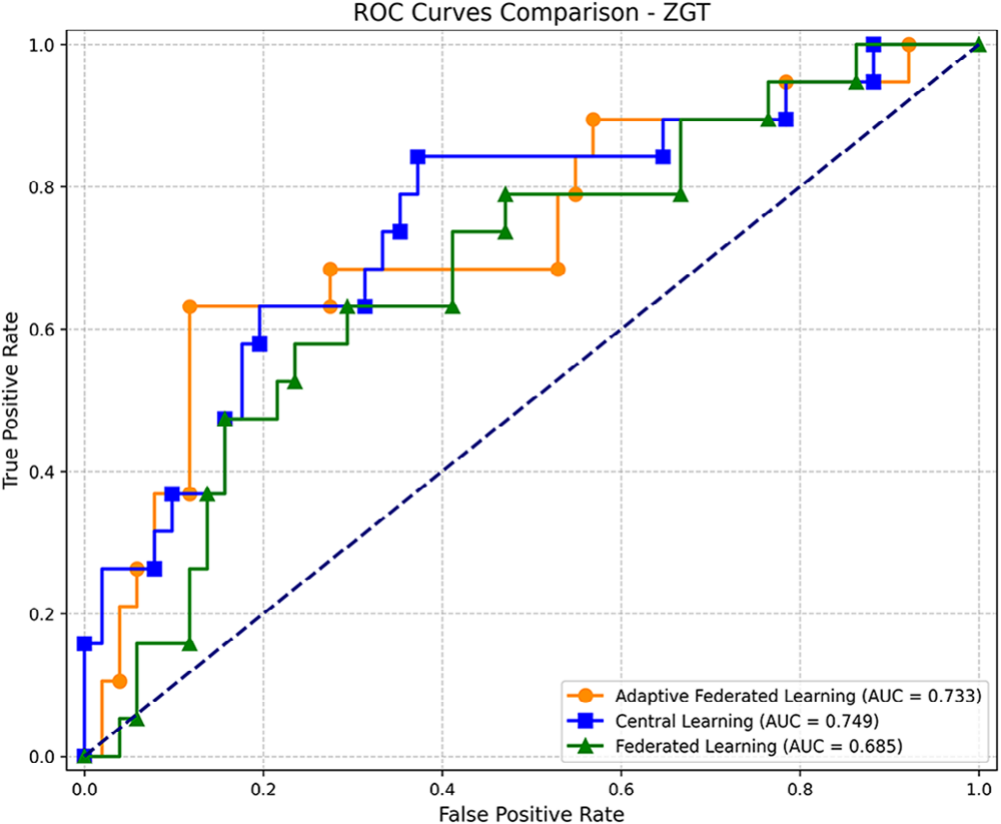
ROC curve comparison for the test center ZGT. ZGT, Ziekenhuisgroep Twente.

Figure 5 further supports these results, presenting the ROC curve comparison for the test center ZGT. The proposed Adaptive FL achieves the highest AUC of 0.733, closely followed by Central Learning (AUC = 0.749), while standard Federated Learning lags behind with an AUC of 0.685. These results demonstrate the superior performance of the proposed method in handling data heterogeneity, improving classification accuracy, and addressing the challenges posed by varying data distributions across centers.

In addition to the global rounds and validation accuracy comparison, Table 4 presents a performance comparison between the proposed adaptive FL method and the traditional standard FedAvg method. The evaluated metrics include specificity, sensitivity (recall), and AUC. The results indicate that our proposed adaptive method outperforms the standard FedAvg approach. To statistically validate the observed improvement in model performance, we conducted a DeLong test^45^ comparing the AUCs between the proposed Adaptive FL method and standard FedAvg. The test yielded a *p*‐value of <0.01, indicating that the improvement from an AUC of 0.685 (FedAvg) to 0.733 (Adaptive FL) on the independent ZGT test set is statistically significant. Similarly, the Central Learning method achieved an AUC of 0.749, which was also significantly higher than FedAvg, with a *p*‐value of <0.007. Additionally, 95% confidence intervals (CIs) for all AUC estimates were computed using bootstrapping with 1,000 resamples, offering robust quantification of uncertainty. The CI for Adaptive FL was [0.70, 0.77], for FedAvg was [0.61, 0.73], with the relatively narrower bounds further supporting the reliability and significance of the performance differences further supporting the observed performance differences.

**TABLE 4:** Performance comparison between adaptive federated learning, central learning, and standard federated learning methods, including AUC confidence intervals and statistical significance versus FedAvg

Model	AUC	95% CI	*p*‐value versus. FedAvg	Sensitivity	Specificity
Adaptive federated learning	0.733	[0.70, 0.77]	< 0.01	0.784	0.786
Standard federated learning	0.685	[0.61, 0.73]	—	0.526	0.784
Central learning	0.749	[0.71, 0.78]	< 0.007	0.895	0.705

Abbreviation: FedAvg, federated averaging.

In addition to evaluating the model on the independent ZGT test center, we also report performance on the internal validation sets from the RUMC and PCNN centers. Table 5 summarizes the AUC, sensitivity, and specificity scores for the RUMC and PCNN validation sets alongside the ZGT test results. The model achieved strong validation performance at both training centers (AUC = 0.76 for RUMC and 0.74 for PCNN), while maintaining high generalization to the unseen ZGT center (AUC = 0.733), confirming the model's robustness across different institutions.

**TABLE 5:** Performance comparison across validation sets from training centers (RUMC and PCNN) and the independent test center (ZGT).

Dataset	AUC	Sensitivity	Specificity
RUMC (Validation)	0.76	0.79	0.75
PCNN (Validation)	0.74	0.77	0.73
ZGT (Test)	0.733	0.784	0.786

Abbreviations: PCNN, Prostate Cancer Neuroendocrine Network; RUMC, Radboud University Medical Center; ZGT, Ziekenhuisgroep Twente.

**FIGURE 6:**
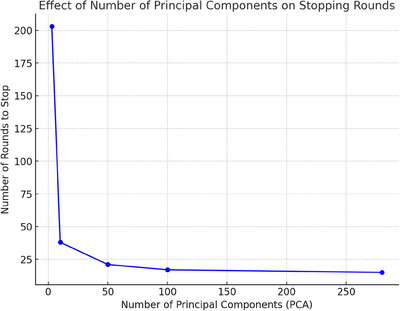
Effect of FIPCA component count on training rounds. FIPCA, federated incremental principal component analysis.

We also have analyzed the relationship between the number of PCs used in FIPCA and the number of global rounds required to stop training in our proposed adaptive method as shown in Figure 6. As observed, when the number of PCs is low (near 0), the number of rounds required for training to stop is at its highest, reaching approximately 200 rounds. However, as the number of PCs increases, the number of stopping rounds rapidly decreases, stabilizing around 25 rounds once the number of principal components reaches approximately 50. This suggests that a moderate number of PCs allows the model to converge much faster, leading to earlier stopping in the training process. This indicates that dimensionality reduction through FIPCA effectively accelerates the training by reducing the number of rounds required for convergence while preserving key information in the dataset.

### 3.2 Resource consumption analysis

We analyzed the resource consumption using an Apple M1 MacBook Pro (2023), featuring an 8‐core CPU and GPU. The device's power consumption ranges between 20 and 30W during full‐load operations. We evaluated the energy consumption (summarized in Table 6) across different setups. The energy consumption can be estimated using Equation 5.

(5) Energy (Wh)=Power (W)×Time (h)

**TABLE 6:** Comparison of training time and energy consumption across methods.

Method	Training time (min)	Estimated energy consumption (Wh)
Standard FedAvg (without FIPCA)	300 (5 h)	100 – 150
FedAvg with FIPCA	15	5 ‐ ‐7.5
Our adaptive FL approach	5	1.6 – 2.5

Abbreviations: FedAvg, federated averaging; FIPCA, Federated Incremental Principal Component Analysis; FL, federated learning.

The proposed Adaptive FL method demonstrates a drastic reduction in training time, memory, and energy consumption. Compared to the standard FedAvg method, it reduces energy consumption by 98%, and by 50% compared to FedAvg with FIPCA. This makes it highly suitable for resource‐constrained federated learning environments. These visualizations collectively confirm the advantages of the adaptive early‐stopping strategy. The method not only reduced computational overhead by minimizing the number of global training rounds but also maintained or enhanced model accuracy, particularly when applied to FIPCA‐transformed datasets. The adaptive approach allowed the model to converge faster, demonstrating its potential for deployment in resource‐constrained FL environments.

To further investigate the contribution of each proposed component, we conducted an ablation study (Table 7). Applying FIPCA alone increased the AUC from 0.685 to 0.726, demonstrating its significant impact on model generalization. In contrast, applying early stopping alone extended training slightly beyond 200 rounds (to 206) with only a marginal AUC improvement (to 0.687), indicating limited standalone benefit. When combined, the full adaptive setup achieved the highest AUC (0.733) and the most efficient training, requiring just 38 rounds and 5 min. These results suggest that FIPCA primarily drives performance gains, while early stopping improves computational efficiency.

**TABLE 7:** Ablation study results showing the contribution of FIPCA and early stopping.

Method	AUC (ZGT Test Set)	Global Rounds	Training Time (minutes)
Standard FedAvg	0.685	200 (fixed)	300
FedAvg + FIPCA only	0.726	200 (fixed)	15
FedAvg + Early stopping only	0.687	206	310
Full Adaptive (FIPCA + Early stopping)	0.733	38	5

## 4 DISCUSSION

The experimental results demonstrate that the proposed FL approach substantially enhances both training efficiency and computational resource usage. This strategy reduces the number of global rounds and helps mitigate overfitting, leading to improved generalization performance, as reflected by a 5% increase in AUC compared to standard FedAvg.

Moreover, the integration of FIPCA effectively reduces data dimensionality, accelerating convergence and optimizing resource efficiency. By aligning data distributions across centers through federated FIPCA, we also reduce the risk of model divergence due to data heterogeneity, enhancing the overall robustness of the global model.

Our results indicate that the proposed method consumes up to 98% less energy than standard FedAvg without FIPCA and 50% less than FedAvg with FIPCA. This energy‐saving aspect is particularly critical in medical settings, where hardware limitations or energy constraints often restrict the deployment of large‐scale federated learning models.

Techniques like partial model sharing, which selectively shares portions of model parameters or gradients, help mitigate privacy risks by reducing data exposure. Approaches such as FLOP keep final layers private while sharing other parts, ensuring privacy and personalization in applications such as COVID‐19 detection.^46^, ^47^ Other methods like cyclic and single weight transfer algorithms aim to improve FL performance but come with trade‐offs, such as biases towards recent clients and increased communication overhead.^48^, ^49^, ^50^, ^51^ Alternative strategies like ensemble learning, which combines multiple model predictions, and split learning, which divides model layers between clients and a central server, enhance generalization and privacy.^52^, ^53^ However, these approaches can increase communication costs and privacy risks, as they often rely on sharing model outputs instead of weights.^54^

The improvements demonstrated by our method not only highlight its practical viability but also show promise for deployment in real‐world, resource‐constrained environments such as healthcare institutions. By minimizing communication rounds and computational demands while preserving privacy, this approach bridges the gap between high‐performance federated learning and energy‐efficient applications in distributed healthcare systems.

Additionally, our method enhances data privacy beyond standard FL approaches by ensuring that only statistical summaries (means and scatter matrices) are shared during FIPCA computation, without any raw data or gradients exchanged. This mitigates critical privacy concerns in medical data sharing and ensures compliance with regulations such as GDPR and HIPAA. By aligning data distributions across centers through federated FIPCA, we also reduce the risk of model inversion attacks that could reconstruct sensitive patient information from shared gradients or model parameters.

Although the results are promising, our experiments were conducted on a specific prostate cancer imaging dataset, and the generalizability of the method to other medical imaging tasks or datasets with different characteristics still requires validation.

Future work should focus on extending this approach to other types of medical imaging data, such as computed tomography (CT) scans or histopathological images, which may present additional challenges related to data dimensionality and heterogeneity. Exploring the applicability of the proposed method in these contexts could further demonstrate its versatility and robustness.

This study focused on evaluating the proposed adaptive framework within the widely adopted FedAvg baseline to demonstrate its generalization and efficiency benefits. While FedAvg remains a strong reference point in federated learning, more recent aggregation strategies such as FedProx, SCAFFOLD, and FedDyn have been proposed to better handle data heterogeneity and client drift. Expanding our evaluation to include comparisons against these methods would provide a broader validation of the proposed improvements. We plan to explore these comparisons as part of future work.

FIPCA effectively reduces data dimensionality and aligns distributions across centers in prostate MR imaging. However, its linear nature may discard subtle features crucial for detecting complex patterns, potentially limiting sensitivity to nuanced abnormalities. The effectiveness of this method should be validated across other dataset types and imaging modalities to ensure robust diagnostic accuracy. So incorporating nonlinear dimensionality reduction techniques, such as federated kernel PCA or federated autoencoders, might capture more complex patterns and relationships in the data, potentially further improving model performance.

In our study the independent test centre (ZGT, 350 cases) operated in inference‐only mode; its images were projected onto the global PCA basis learned from the two training centres (RUMC, PCNN) without contributing local statistics. We recommend the same strategy for sites with very small cohorts, fewer than ≈50 cases where scatter‐matrix estimates become noisy, late joiners after the global basis is fixed (with updates introduced in planned releases), centres with persistent data‐quality issues, and institutions whose strict privacy policies or unreliable connectivity prevent even summary‐statistic exchange.

Although our experiments use voxel‐level features, the FIPCA step is mathematically agnostic to feature type. It operates solely on first‐ and second‐order statistics—namely, the mean vector and scatter matrix—computed from any numerical feature space that is consistent across clients. As such, radiomic descriptors, handcrafted texture features, or neural network embeddings could be used interchangeably without altering the algorithm, provided the feature dimensionality is uniform across sites. However, radiomic features may require additional harmonization steps to ensure consistency across institutions due to their sensitivity to variations in imaging protocol, segmentation, and preprocessing. Extending this framework to alternative feature representations represents a promising direction for future research.

Finally, the choice of the number of principal components is a critical hyperparameter that affects both computational efficiency and the model's ability to capture essential data variance. While our study selected 10 principal components to balance these factors, adaptive methods for selecting the optimal number could further enhance performance. Techniques, like explained variance thresholds or cross‐validation, could help automatically determine the most informative number of components for different datasets.

## 5 CONCLUSION

The proposed method improves the standard FedAvg algorithm by applying FIPCA for dimensionality reduction, aggregating local means and scatter matrices to compute a global model while maintaining privacy. This approach reduces data heterogeneity, enhances computational efficiency, and preserves privacy by sharing only statistical summaries. Additionally, an adaptive early stopping mechanism based on aggregated client validation loss minimizes global rounds, speeding up convergence without sacrificing accuracy.

Experimental results on the PI‐CAI dataset show a 98% reduction in energy consumption, with global rounds reduced from 200 to 38 and AUC improved from 0.68 to 0.73 (95% CI 0.70 – 0.77). The method also improves robustness to data heterogeneity, making it suitable for resource‐constrained environments, where computational efficiency and resource optimization are crucial for large‐scale FL approaches.

## CONFLICT OF INTEREST STATEMENT

The authors have no conflicts to disclose.

## Data Availability

The dataset used in this study is publicly available through the Prostate Imaging Cancer AI (PI‐CAI) challenge^42^ and can be accessed online. The code for our FL framework is available on GitHub at https://github.com/npiran/federated_learninggithub.com/npiran/federated‐learning for reproducibility.
